# TAPB and RSB protects cardiac diastolic function in elderly patients undergoing abdominopelvic surgery: a retrospective cohort study

**DOI:** 10.7717/peerj.9441

**Published:** 2020-07-02

**Authors:** Chao Gong, Shitong Li, Xiaojing Huang, Lianhua Chen

**Affiliations:** Department of Anesthesiology, Shanghai General Hospital of Nanjing Medical University, Nanjing Medical University, Shanghai, China

**Keywords:** Diastolic function, Transesophageal echocardiography (TEE), Sevoflurane, Transversus abdominis plane block and rectus sheath block (TAPB and RSB)

## Abstract

**Background:**

Diastolic dysfunction, an early manifestation and clinical symptom of heart failure with preserved ejection fraction, can be influenced by various anesthesia management strategies. Trans-esophageal echocardiography was used to undertake to assess left ventricular diastolic function during anesthesia maintenance using sevoflurane alone and sevoflurane combining with transversus abdominis plane block and rectus sheath block in elderly patients with diastolic dysfunction undergoing abdominopelvic surgery.

**Methods:**

Thirty-eight patients were divided into two groups in this retrospective study, sevoflurane and sevoflurane combining with TAPB and RSB according to employing different anesthesia maintenance schemes. The parameters HR, MAP, CVP, E, A, E/A, e, a, e/a, and E/a were obtained immediately after anesthesia induction hemodynamics stability (HR1, MAP1, CVP1, E1, A1, E1/A1, e1, a1, e1/a1, and E1/a1) and 1 hour later (HR2, MAP2, CVP2, E2, A2, E2/A2, e2, a2, e2/a2, and E2/a2).

**Results:**

Transmitral diastolic Doppler flow characteristics illustrated E/A significant decreases in the S group but increases in the ST group (*p* = 0.02 < 0.05) 1 hour after anesthesia induction hemodynamic stability. Tissue Doppler imaging characteristics showed a more significant increase e/a (*P* = 0.005 < 0.05) and decreases in a value (*p* = 0.009 < 0.05) in the ST group 1 hour after anesthesia induction hemodynamics stability.

**Conclusions:**

Maintaining anesthesia with sevoflurane combining with TAPB and RSB was more suitable for protecting cardiac diastolic function than sevoflurane alone in elderly patients with diastolic dysfunction undergoing open abdominal and pelvic surgery.

## Introduction

Cardiovascular disease is the primary cause of death worldwide and heart failure (HF) and atrial fibrillation continue to increase especially in the elderly (Braunwald, 1997). In HF patients, myocardial dysfunction may be caused by ischemia or hypertension, which are the most common complications. Approximately half of patients with HF without systolic dysfunction have preserved ejection fraction (HFpEF) (Maxwell, Konoske & Mark, 2016). The five-year survival rate of patients with HFpEF is approximately 50% (Dunlay, Roger & Redfield, 2017).

Diastolic dysfunctioly is an early manifestation of HFpEF, or a clinical symptom of HFpEF and is defined as that ventricle can not be filled to an adequate end-diastolic volume during the diastolic phase (Matyal et al., 2011). Perioperative evidence suggests that HFpEF is an independent postoperative morbidity and mortality predictor for patients undergoing major noncardiac surgery (Shillcutt et al., 2014). To enhance patient safety, appropriate anesthesia strategy of monitoring diastolic function during the perioperative stage is crucial for anesthesiologists. Meanwhile, echocardiography is an important method to characterize diastolic function and particularly transesophageal echocardiography (TEE) is a conventional and accurate method to inspect cardiac diastolic function.

Different anesthetics cause various degrees of inhibition of diastolic function (Sarkar, GuhaBiswas & Rupert, 2010; Ammar et al., 2016; Lee et al., 2016). Thus, for patients with cardiovascular disease, it is vital to implement appropriate anesthesia maintenance strategies to substantially reduce the impact on heart function as much as possible.

General anesthesia combined with local anesthesia, which can be precisely implemented by the application of intraoperative ultrasound, has been widely proven to decrease surgical stimulation, reduce the use of general anesthetics and opioid, and ensure hemodynamic stability perioperative (Tsuchiya et al., 2012). Transversus abdominis plane block and rectus sheath block (TAPB and RSB) has been widely effective used in balance anesthesia and postoperative analgesia of abdominopelvic surgery, which can block the regulation of sensory nerve at the anterior abdominal well. Thus, TAPB and RSB can reduce the stress, inflammation and the consumption of other anesthetics. While general anesthesia combined with TAPB and RSB has been widely applied in clinical practice, few study reports on the effect of sevoflurane combining with TAPB and RSB on diastolic function in elderly with diastolic dysfunction undergoing non-cardiac surgery. Therefore, this retrospective study was conducted to assess different effects on left ventricular (LV) diastolic function in elderly patients with diastolic dysfunction undergoing abdominopelvic surgery between sevoflurane alone and sevoflurane combining with TAPB and RSB by using TEE detection. Because of the advantages of TAPB and RSB, we hypothesized that sevoflurane combining with TAPB and RSB might be more advantageous than sevoflurane in diastolic dysfunction patients.

## Materials & Methods

### Subjects

This was a non-randomized, retrospective clinical observational study, based on previous perioperative management date (between June 2015 and December 2017), and was approved by our hospital clinic research committee. The need for consent was waived for this study, as data was previously collected as part of regular hospital procedures and all patients previously consented to data collection prior to surgery. Patients were contacted in May 2018 before enrolling in the current retrospective study to consent to the use of their data for the purpose of this research. It included 38 patients who were scheduled for grade 3–4 through open abdominal and pelvic surgery (Table 1) with American Society of Anesthesiologists physical status II–III, age >60 years (70.21 ± 6.94), and demonstrated a sinus rhythm, mitral valvular (MV) e velocity <10 cm/s, and averaged LV E/e >9 cm/s by TEE evaluation after anesthesia induction hemodynamics stability indicating diastolic dysfunction (Ammar et al., 2016; Matyal et al., 2011). The hemodynamics stability was that hemodynamic values were maintained between 15% pre-induction baseline values.

**Table 1:** Patient characteristics and surgical data.

	Sevoflurane, (*n* = 19)	Sevoflurane combining + TAPB and RSB, (*n* = 19)
Age, years (M ± SD)	68.89 ± 5.23	71.53 ± 8.24
Male, n	10	9
Female, n	9	10
ASA score III, n	1	0
ASA score II, n	18	19
Duration of surgery (hours)	3.16 ± 1.32	3.23 ± 0.81
Blood glucose (mmol/L)	5.39 ± 1.06	6.01 ± 1.49
Triglyceride (mmol/L)	1.14 ± 0.44	1.13 ± 0.52
Cholesterol (mmol/L)	3.96 ± 1.23	3.77 ± 0.93
Hypertension, n	4	5
Diabetes mellitus, n	3	0
Gastrointestinal surgery, n	11	15
Urology surgery, n	7	2
Gynecologic surgery, n	1	2

**Notes.**

TAPB and RSB, transversus abdominis plane block and rectus sheath block.

Patients who met these criteria during surgeries from June 2015 to December 2017 were enrolled, following approval by our hospital ethics committee (2018KY173) and registered at Chinese Clinical Trial Registry (ChiCTR1900021671). Patients with the following characteristics were excluded: left ventricular ejection fraction (LVEF) <60% preoperative by trans-thoracic echocardiography, preexisting valvular disease, pericardia disease, and serious LV hypertrophy, infection on the puncture site, history of allergy to local anesthetics, preexisting neurological disorders, and/or coagulation system disorders (recent bleeding from esophageal varicose veins or hypocoagulability with prothrombin time <20%).

### Anesthesia conditions

Because of complicated operations, patients underwent routine monitoring, including 5-lead electrocardiography and pulse oximetry. A central venous line and femoral arterial line were installed after anesthesia induction. Anesthesia was induced by propofol 1.5 mg/kg (Fresenius Kabi AB, Uppsala, Sweden), fentanyl 4 µg/kg (Renfu Pharmaceutical Co. Ltd., Yichang, China), and rocuronium 1 mg/kg (N.V. Organon, Oss, Netherland); it was maintained by either sevoflurane combining with fentanyl 2 µg/kg/h after skin incision (S group) or sevoflurane combining with TAPB and RSB (ST group). During the surgery, the concentration of sevoflurane, with 1 L/min 80% oxygen and air, was maintained stable MAC level by adjusting to maintain bispectral index (BIS) range between 40 and 50, and hemodynamic stability in both groups. Rocuronium was added as need 0.2 mg/Kg. Cardiac preload monitored by central venous pressure (CVP) was kept within the normal range (5–12 cm H_2_O) by intravenous fluid infusion.

Bilateral transversus abdominis plane block and rectus sheath block was implemented by visible ultrasound guiding after anesthesia induction and inserting the trans-esophageal ultrasound probe as the description (Xia et al., 2020). The local anesthetic of transversus abdominis plane block was injected between the fascial planes of the internal oblique and transversus abdominis muscles causing the anesthetic diffusion between the internal oblique and transversus abdominis. For the rectus sheath block, local anesthetic was injected into anterior to the posterior rectus sheath causing distribution between the muscle and posterior plane. The combination local anesthetic of 1% Lidocaine (ZhaoHui Pharmaceutical Co. Ltd., shanghai, China) and 0.375% ropivacaine (AstraZeneca AB, Sweden) 10 ml was implemented at each injection sites of four locations.

Heart rate (HR) and mean arterial pressure (MAP) were kept stability at an appropriate level by treating with atropine (HR was below 50 bpm), *β*1-adrenergic receptor antagonist esmolol (HR was above 100 bpm), *α*1-adrenergic receptor agonist phenylephrine (MAP was below 60 mmHg) and calcium channel blocker perdipine (MAP was above 120 mmHg). The values were obtained after anesthesia induction (HR_1_, MAP_1_), thus representing baseline, and 1 h after that (HR_2_, MAP_2_), thus illustrating hemodynamic variation at 1 h after induction hemodynamics stability to exclude the effect of after-load.

### Diastolic function

TEE was performed according to the practice guidelines of perioperative TEE established by the American Society of Anesthesiologists and European Association of Cardiovascular, and was executed by the same experienced anesthesiologist as the above procedures. After anesthesia induction and endotracheal intubation, an ultrasonic probe was inserted in the mid-esophageal with four-chamber view to ensure that the ultrasound beam was parallel to the long-axis movement of the mitral annular and that the sample volume was placed between the tips of the mitral valve leaflets. And relevant diastolic function parameters were collected after anesthesia induction hemodynamics stability and 1 h later respectively.

With a pulsed-wave technique, some Doppler parameters were obtained, including the transmitral peak flow velocity of early diastolic filling (E) and the transmitral peak flow velocity of late diastolic filling (A) (Shanewise et al., 1999; Nagueh et al., 2009). In addition to blood stream velocity, mitral annular tissue velocity can be recorded by spectral Tissue Doppler Imaging (TDI). By this method, the sample volume was assessed at the level of mitral lateral annular from the 4-chamber view, and parameters were measured based on mitral lateral annular movement in early diastole (e) and in late diastole (a). Concurrently, the E/A, e/a, and E/e ratios were obtained.

### Statistical methods

**Figure 1:**
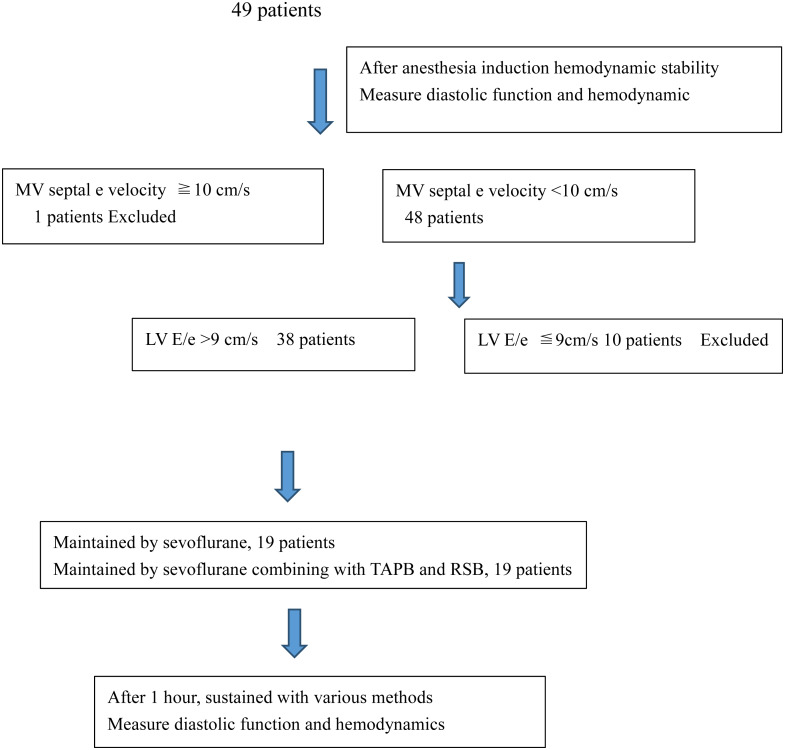
The grouping situation depends on collected dates.

Although Our research was retrospective, we evaluated the reasonableness of the sample size based on the the results of the main indicator e/a (SD_sev_ = 0.3; Mean_sev_ =0.85; Mean_sev+TAPB_ = 1.19). With a power of 0.8 and a significance level of 0.05, at least 17 patients were need in each group. Assuming a dropout rate of 10%, 19 patients were selected for each group. Date were collected in Microsoft excel and assessed by using SPSS version 22.0 (IBM Corporation, Armonk, NY, USA). As shown in Fig. 1, we divided patients into two groups, sevoflurane group (S group) and sevoflurane combining with TAPB and RSB group (ST group), according to their strategies for maintaining anesthesia. Continuous variables were presented as Mean ± SD (standard deviation) and compared by using a linear mixed model between the 2 groups and using a paired *t*-test in group. For all assessments, a probability value of *p* < 0.05 (2-sided) was considered statistically significant.

## Results

### Baseline characteristics

The baseline characteristics of our patients were presented in Table 1. The mean age of the 38 consecutive study patients (19 men and 19 women) was 70.21 ± 6.94 years. Based on American Society of Anesthesiologists (ASA) classification, 37 patients were class II and 1 patient was class III. The mean duration time of surgery was 3.20 ±1.08 h. Patients exhibited the following complications: hypertension (9) and Diabetes Mellitus (3) and medications were continued until the morning of surgery. Of the 38 patients, 19 were administered sevoflurane and 19 were administered sevoflurane combining with TAPB and RSB during anesthesia maintenance.

The anesthesia maintenance management informations were shown in Table 2. ST group decreased the use of sevoflurane indicated with maintaining minimum alveolar concentration (MAC) (1.22 ± 0.13 vs. 0.77 ± 0.67), however, there were no statistical significance. And there were no significantly difference in infusion volume, blood loss, urine output, and vasoactive agents between S group and ST group from anesthesia induction to 1 h later.

**Table 2:** The anesthesia maintenance management informations.

	Sevoflurane, (*n* = 19)	Sevoflurane + TAPB and RSB, (*n* = 19)	*P*
Infusion volume (ml)	638.68 ± 89.43	618.42 ± 79.49	0.958
Blood loss (ml)	82.53 ± 14.57	77.53 ± 18.16	0.413
Urine output (ml)	443.63 ± 123.76	444.05 ± 116.64	0.435
MAC (sevoflurane)	1.22 ± 0.13	0.77 ± 0.67	0.146
Atropine (mg)			
Number of patients used	0.03 ± 0.10 (2/19)	0.02 ± 0.06; (2/19)	0.366
Esmolol (mg)			
Number of patients used	1.05 ± 3.15 (2/19)	1.05 ± 2.68; (3/19)	0.88
Phenylephrine (µg)			
Number of patients used	0 (0/19)	0 (0/19)	–
Perdipine (mg)			
Number of patients used	0.08 ± 0.19 (3/19)	0.05 ± 0.15; (2/19)	0.35

**Notes.**

TAPB and RSB, transversus abdominis plane block and rectus sheath block.

MAC minimum alveolar concentration

### Hemodynamic features after anesthesia induction hemodynamics stability (T_1_) and 1 h later (T_2_)

There were no statistical differences in HR_1_ (68.37 ± 11.55 vs. 68.05 ± 11.20) and MAP_1_(90.32 ± 15.47 vs. 91.68 ± 11.79); HR_2_ (68.79 ± 10.14 vs. 67.47 ± 9.63) and MAP_2_ (91.84 ± 12.13 vs. 88.21 ± 11.02); CVP_1_ (8.26 ± 1.24 vs. 8.26 ± 1.41) and CVP_2_ (8.47 ± 1.58 vs. 8.53 ± 1.57) between S group and ST groups. Results were shown in Table 3.

**Table 3:** Hemodynamic features in sevoflurane and sevoflurane + TAPB and RSB groups with diastoliccdysfunction undergoing noncardiac surgery.

	T1	T2	Pgroup × times
MAP (mmHg)			0.731
sev	90.32 ± 15.47	91.84 ± 12.13	
sev + TAPB + RSB	91.68 ± 11.79	88.21 ± 11.02	
HR (bpm)			0.787
sev	68.37 ± 11.55	68.79 ± 10.14	
sev + TAPB + RSB	68.05 ± 11.20	67.47 ± 9.63	
CVP (cmH_2_O)			0.946
sev	8.26 ± 1.24	8.47 ± 1.58	
Sev + TAPB + RSB	8.26 ± 1.41	8.53 ± 1.57	

**Notes.**

Sev sevoflurane TAPB + RSB transversus abdominis plane block and rectus sheath block MAP mean arterial pressure HR heart rate; CVP central venous pressure T1 immediately after anesthesia induction hemodynamic stability as a baseline T2 1 hour after T1

### Transmitral diastolic Doppler flow characteristics after anesthesia induction hemodynamics stability (T_1_) and 1 h later (T_2_)

The TEE probe was securely placed in all patients without esophageal bleeding. We analyzed the parameters, shown in Table 4, regarding diastolic function investigated by transmitral diastolic doppler flow, comparing S group with ST group after anesthesia induction hemodynamics stability (T_1_) and 1 h later (T_2_). ST group caused some improvement of LV diastolic function 1 h after T1, as the evidence by significant decreases in E/A in S group but increases in ST group (1.07 ± 0.38 vs. 1.36 ± 0.33, *p* = 0.022 <0.05). TEE images were shown in supplementary materials 1 and 3.

**Table 4:** Transmitral diastolic Doppler flow characteristics in sevoflurane and sevoflurane + TAPB and RSB groups with diastolic dysfunction undergoing noncardiac surgery.

	T_1_	T_2_	P_group×times_
E, cm.s^−1^			0.941
sev	75.61 ± 30.78	80.36 ± 38.80	
sev + TAPB + RSB	77.72 ± 30.61	77.10 ± 20.70	
A, cm.s^−1^			0.106
sev	66.70 ± 33.65	77.77 ± 35.43	
sev + TAPB + RSB	61.04 ± 24.69	59.40 ± 20.16	
E/A^*^			0.022 <0.05
sev	1.18 ± 0.26	1.07 ± 0.38	
sev + TAPB + RSB	1.35 ± 0.45	1.36 ± 0.33	

**Notes.**

Sev sevoflurane TAPB + RSB transversus abdominis plane block and rectus sheath block E transmitral peak flow velocity of early diastolic filling A teansmitral peak flow velocity of late diastolic filling T_1_ immediately after anesthesia induction hemodynamic stability as a baseline T_2_ 1 hour after T_1_

* Represent *p* < 0.05 between the two groups (linear mixed model).

### Tissue Doppler imaging characteristics after anesthesia induction hemodynamics stability (T_1_) and 1 h later (T_2_)

Several measurements (e, a, e/a, E/e) were implemented simultaneously to investigate diastolic function by tissue Doppler shown in Table 5. However, unlike transmitral diastolic Doppler flow characteristics, both S group and ST group caused some improvement of left ventricular diastolic function according to the TDI characteristics. Improvement of LV diastolic function was greater in ST group than in S group 1 h after anesthesia induction stability, evidenced by more significant increase e/a (0.85 ± 0.30 vs. 1.19 ± 0.43, *P* = 0.005 <0.05) and significant decreases in a value in ST group (7.12 ± 1.67 vs. 5.45 ± 1.48, *p* = 0.009 <0.05). At the same time, some parameters, both S group and ST groups increasing in e value (5.71 ± 1.31 vs. 6.25 ± 2.25) and decreasing in E/e (14.25 ± 6.42 vs. 13.85 ± 5.85) 1 h after anesthesia induction stability, indicated diastolic function improvement, however, there were no statistical significance. TEE images were shown in supplementary materials 2 and 4.

**Table 5:** Tissue Doppler imaging characteristics of in sevoflurane and sevoflurane + TAPB and RSB groups with diastolic dysfunction undergoing noncardiac surgery.

	T_1_	T_2_	P_group×times_
e, cm.s^−1^			0.386
sev	5.13 ± 1.30	5.71 ± 1.31	
sev + TAPB + RSB	5.31 ± 1.49	6.25 ± 2.25	
a^**^, cm.s^−1^			0.009 <0.01
sev	6.31 ± 1.68	7.12 ± 1.67	
sev + TAPB + RSB	5.58 ± 1.72	5.45 ± 1.48	
e/a^**^			0.005 <0.01
sev	0.77 ± 0.33	0.85 ± 0.30	
sev + TAPB + RSB	1.03 ± 0.35	1.19 ± 0.43	
E/e			0.989
sev	15.19 ± 6.06	14.25 ± 6.42	
sev + TAPB + RSB	15.55 ± 7.45	13.85 ± 5.85	

**Notes.**

Sev sevoflurane TAPB + RSB transversus abdominis plane block and rectus sheath block e mitral lateral annulus movement in early diastole a mitral lateral annulus movementin in late diastole T_1_ immediately after anesthesia induction hemodynamic stability as a baseline T_2_ 1 hour after T_1_

** Represent *p* < 0.01 between the two groups (linear mixed model).

## Discussion

HFpEF is a symptom of a heart that has near-normal ventricular systolic function with ventricular diastolic dysfunction (Borlaug, 2014). Diastolic function can be measured in various ways and the gold standard is collection by echocardiography. Notably, TEE is an accurate and feasible instrument to measure heart function for perioperation. Transmitral inflow velocities (E, A) obtained by Doppler may be impacted by cardiac blood volume, LA pressure increase creating pseudo-normal in spite of using the anesthesia machine “pop-off” valve to gain the appropriate inspiratory pressure, thus, combining measuring mitral leaflet movements with tissue Doppler, is a more precise method to evaluate diastolic function (Shanewise et al., 1999). To further improve the accuracy of diastolic dysfunction measurement, implementing additional measurements (E/A, e/a, E/e) is necessary (Ammar et al., 2016; Lee et al., 2016; Sarıet al., 2016; Matyal et al., 2011).

Different diastolic function foundations may exhibit various statuses with diverse anesthesia management protocols. In patients without diastolic dysfunction before operation, inhaled anesthetics could impair left ventricular diastolic function (Sarkar, GuhaBiswas & Rupert, 2010). However, many clinical studies have shown that patients with left ventricular diastolic dysfunction before anesthesia could be improved by using isoflurane, desflurane, or sevoflurane owing to reducing the after-load of the left ventricle to achieve a normal filling pattern (Sarkar, GuhaBiswas & Rupert, 2010). Ammar, A. reported that isoflurane was favourable over propofol on diastolic dysfunction (Ammar et al., 2016). It was possible that anesthetic agents could alter sarcoplasmic reticulum calcium homeostasis at the sarcoplasmic reticulum, a pivotal component of normally myocardial relaxation (Pagel et al., 1993).

This conclusion was partially confirmed in our patients maintained by sevoflurane. In our investigation, some diastolic function parameters, although showed no notable statistical change in both groups, indicated different influence maintenance anesthesia with sevoflurane along, some tendency (E_1_ <E_2_, e_1_ <e_2_, E_1_/e_1_ >E_2_/e_2_) showed improvement diastolic function and some tendency (A_1_<A_2_) showed deteriorating diastolic function, but all measurements showed improvement diastolic function with sevoflurane combining with TAPB and RSB maintaining. Meanwhile, using sevoflurane combining with TAPB and RSB to implement anesthesia maintenance could improvement diastolic function better than using sevoflurane alone, confirmed by more accurate and appropriate measurements (E/A, e/a, E/e) with significantly statistical difference. This effect might be attributed to the decreased volume of inhalation anesthetic and combined with local anesthesia.

Thus, our results underlined the importance of TAPB and RSB. Due to the development of ultrasound technology, many regional anesthesia can be securely and visually implemented and has been used daily in clinical anesthesia and postoperative analgesia. TAPB and RSB could blunt non-nociceptive and nociceptive stimulation, and anti-inflammation (Kuchálik et al., 2017), sevoflurane combining with TAPB and RSB exhibited less interference on diastolic function in elderly patients with preexisting diastolic dysfunction.

Hemodynamics, variation of cardiac preload and afterload, was not a neglectable factor affecting diastolic function measurement, regardless of increase or decrease (Ammar et al., 2016). Accordingly, managing pro-load by monitoring CVP and managing after-load by controlling HR and MAP were applied to reduce the impact of hemodynamics on diastolic function measurement.

Studies have shown that general anesthesia and regional anesthesia have anti-inflammatory effects (Kuchálik et al., 2017). Our findings might be related with this aspect. However, general anesthesia was not better for diastolic dysfunction than regional anesthesia in our observation. Thus, we will perform additional research to resolve the anti-inflammatory mechanism of TAPB and RSB. A major limitation of the study was its retrospective nature, which complicates analysis of the relationship between diastolic function and anesthesia strategy. Thus, we will conduct a larger scale randomized controlled study to confirm our findings.

## Conclusions

Generally, for optimal cardiac diastolic function, it is better to maintain anesthesia with sevoflurane combining with regional anesthesia than with sevoflurane along in elderly patients with diastolic function.

##  Supplemental Information

10.7717/peerj.9441/supp-1 Data S1 Raw data

10.7717/peerj.9441/supp-2 Supplemental Information 2 The echocardiography between S group and ST group

## Abbreviations

A: trans-mitral peak flow velocity of late diastolic filling,
a: mitral lateral annular movement in late diastole
ASA: American Society of Anesthesiologists
BIS: bispectral index
CVP: central venous pressure
E: trans-mitral peak flow velocity of early diastolic filling
e: mitral lateral annular movement in early diastole
EF: ejection fraction
HR: heart rate
HF: heart failure
HFpEF: HF without systolic dysfunctionhave preserved ejection fraction
LV: left ventricular
MAC: minimum alveolar concentration
MAP: mean arterial pressur
MV: mitral valvular
S group: sevoflurane group
ST group: sevoflurane combining with transversus abdominis plane block and rectus sheath block group
TAPB and RSB: transversus abdominis plane block and rectus sheath block
TEE: trans-esophageal echocardiography
TDI: Tissue Doppler Imaging

## Additional Information and Declarations

### Data Availability

The following information was supplied regarding data availability: The raw measurements are available in a Supplemental File.

### Clinical Trial Registration

The following information was supplied regarding Clinical Trial registration: ChiCTR1900021671.
