# Supplementary material for: TAPB and RSB protects cardiac diastolic function in elderly patients undergoing abdominopelvic surgery: a retrospective cohort study
Source: PeerJ. 2020 Jul 2;8:e9441. doi: 10.7717/peerj.9441 (PMC7335498; doi:10.7717/peerj.9441)
Supplement: Supplemental Information 2 [file peerj-08-9441-s002.pdf]

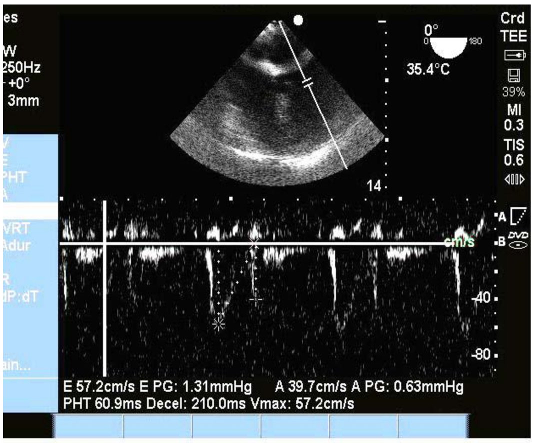

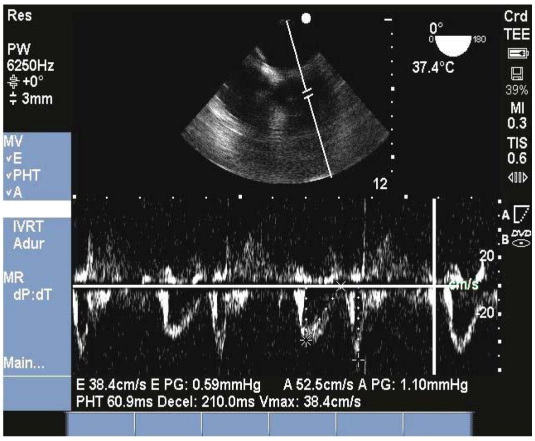

A

B

S1. Diastolic function (E and A) of the sevoflurane group after anesthesia induction hemodynamic stability (A) and at 1 hour later (B).

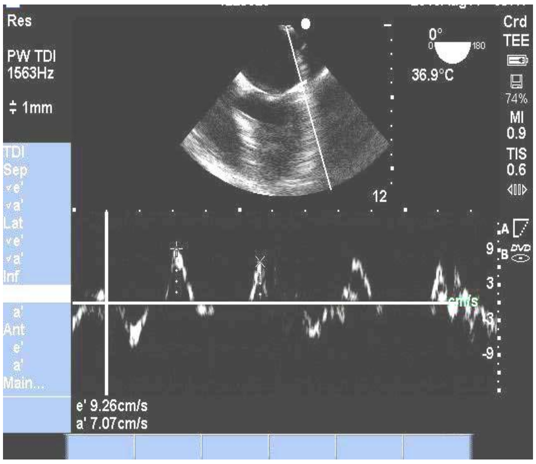

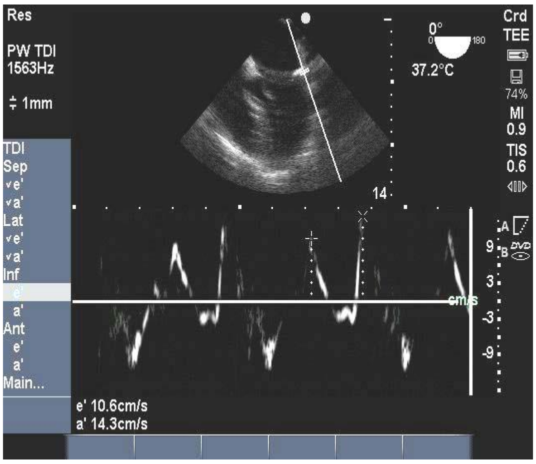

C

D

S2. Diastolic function (e and a) of the sevoflurane group after anesthesia induction hemodynamic stability (C) and at 1 hour later (D).

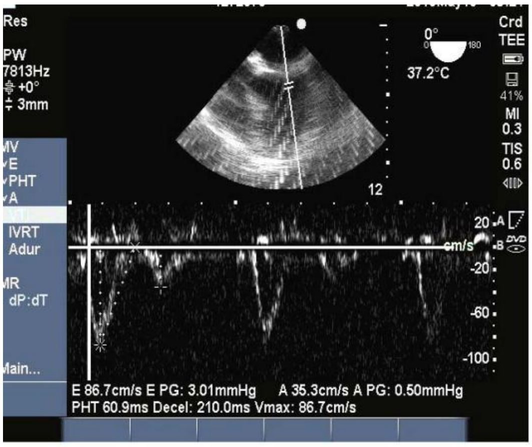

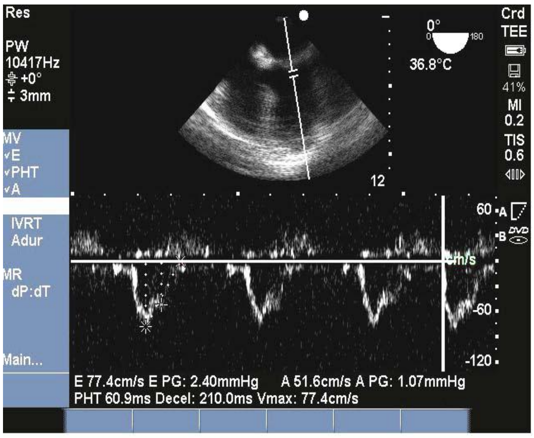

E

F

S3. Diastolic function (E and A) of the sevoflurane + TAPB and RSB group after anesthesia induction hemodynamic stability (E) and at 1 hour later (F).

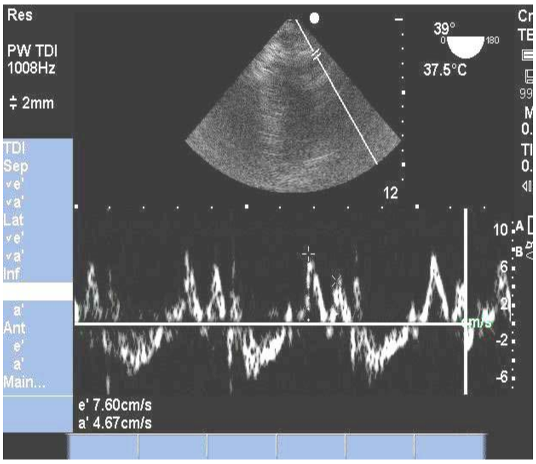

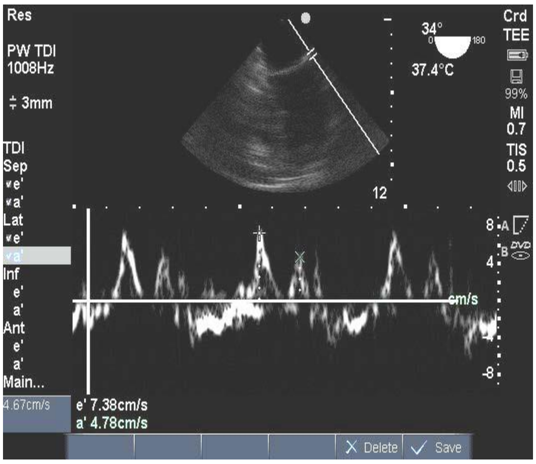

G

H

S4. Diastolic function (e and a) of the sevoflurane + TAPB and RSB group after anesthesia induction hemodynamic stability (G) and at 1 hour later (H).
